# Neural Mechanisms of Hearing Recovery for Cochlear-Implanted Patients: An Electroencephalogram Follow-Up Study

**DOI:** 10.3389/fnins.2020.624484

**Published:** 2021-02-05

**Authors:** Songjian Wang, Meng Lin, Liwei Sun, Xueqing Chen, Xinxing Fu, LiLi Yan, Chunlin Li, Xu Zhang

**Affiliations:** ^1^School of Biomedical Engineering, Capital Medical University, Beijing, China; ^2^Beijing Key Laboratory of Fundamental Research on Biomechanics in Clinical Application, Capital Medical University, Beijing, China; ^3^Key Laboratory of Otolaryngology Head and Neck Surgery, Beijing Tongren Hospital, Beijing Institute of Otolaryngology, Ministry of Education, Beijing, China

**Keywords:** cochlear implant, independent component analysis, auditory function remodeling, phase lag index, functional connection, event-related potential

## Abstract

**Background:**

Patients with severe profound hearing loss could benefit from cochlear implantation (CI). However, the neural mechanism of such benefit is still unclear. Therefore, we analyzed the electroencephalogram (EEG) and behavioral indicators of auditory function remodeling in patients with CI. Both indicators were sampled at multiple time points after implantation (1, 90, and 180 days).

**Methods:**

First, the speech perception ability was evaluated with the recording of a list of Chinese words and sentences in 15 healthy controls (HC group) and 10 patients with CI (CI group). EEG data were collected using an oddball paradigm. Then, the characteristics of event-related potentials (ERPs) and mismatch negative (MMN) were compared between the CI group and the HC group. In addition, we analyzed the phase lag indices (PLI) in the CI group and the HC group and calculated the difference in functional connectivity between the two groups at different stages after implantation.

**Results:**

The behavioral indicator, speech recognition ability, in CI patients improved as the implantation time increased. The MMN analysis showed that CI patients could recognize the difference between standard and deviation stimuli just like the HCs 90 days after cochlear implantation. Comparing the latencies of N1/P2/MMN between the CI group and the HC group, we found that the latency of N1/P2 in CI patients was longer, while the latency of MMN in CI users was shorter. In addition, PLI-based whole-brain functional connectivity (PLI-FC) showed that the difference between the CI group and the HC group mainly exists in electrode pairs between the bilateral auditory area and the frontal area. Furthermore, all those differences gradually decreased with the increase in implantation time.

**Conclusion:**

The N1 amplitude, N1/P2/MMN latency, and PLI-FC in the alpha band may reflect the process of auditory function remodeling and could be an objective index for the assessment of speech perception ability and the effect of cochlear implantation.

## Introduction

Cochlear implantation (CI) is a well-established means to restore normal hearing for people with severe hearing impairment. Although the CI technology continues to advance, there remains a significant variability in speech perception performance among CI users (Paiva et al., 2016). Following CI implantation, the reacquisition of speech recognition ability is considered as the aim of CI rehabilitation (Mareike Finke et al., 2016). For CI users, behavioral measures such as the category of auditory performance score and speech intelligibility rating (SIR) score are the primary methods to evaluate speech perception ability and the effect of implantation. However, it is hard to apply these methods to people with prelingual deafness or prolonged auditory deprivation and children, since they are unable to complete the speech tests because of their poor communication and cognitive function (Mareike Finke et al., 2016). Accordingly, objective methods are needed to evaluate the effect of cochlear implantation.

Previous studies in CI users employed the electroencephalogram (EEG) to assess the status of the central auditory cortex. Event-related potentials (ERPs), also termed as long latency potentials, were previously used as indicators for auditory processing. The N1, P2, and mismatch negative (MMN) are the three widely studied components among the human auditory-evoked potentials. N1 occurs in the 60–150-ms interval after stimulus onset and is followed by the P2, a positive deflection peak at a latency around 150–250 ms (Roth et al., 1976). In addition, the MMN reflects a preattentive auditory-evoked potential (AEP) that occurs 100–300 ms following sound onset and is characterized by a front-central negativity elicited during any acoustically discriminable “deviant” sound within a regular stream of “standard” stimuli (C.W. Ponton et al., 2000; Näätänen et al., 2007; Moberly et al., 2016). N1, P2, and MMN are believed to be potential candidates in developing models of auditory information processing.

In our study, the first aim is to confirm the recovery of speech recognition and auditory perception in CI users. Previous studies suggest that ERPs can better assess the hearing recovery of patients with CI, compared with the auditory brainstem response (ABR), such that ERPs can reflect the brain activity induced by the auditory signal at the cortical level. In a seminal review on the auditory N1 wave (Roth et al., 1976), the N1 characteristic waveform detected at the central frontal electrode may originate from the auditory cortex on the dorsal surface of the temporal lobe. N1 activity is also related to the detection and discrimination of changes in the auditory environment. Besides, adult N1 would be sensitive to onset features, such as the slope and amplitude of the rise and fall of the auditory stimulus, which correlates with detection. The amplitude of N1 increases as the intensity of the standard stimulus increases, showing sensitivity to sound intensity in CI users. Previous research found that the amplitude of N1 significantly increases 1 year after CI implantation (Tremblay et al., 2014; Paiva et al., 2016). These changes may reflect the cortical reorganization of the auditory cortex and the restoration of binaural function in patients with CI.

Thus, we evaluated the change of N1 amplitude in the speech recognition experiment at different implantation stages to explore the speech recognition ability of CI users by comparing brain signals induced by standard stimuli and deviation stimuli.

The second aim is to investigate how the N1 and P2 representations changed in CI users with the increase of implantation time. Similar to N1, the P2 component also has clinical significance in evaluating the speech recognition function of CI users. P2 is thought to index some aspects of stimulus classification, reflecting processes of attentional allocation, perceptual learning, and event memory (Arnott et al., 2011; Paiva et al., 2016). It has also been suggested that its front-central prominence is related to the inhibitory process caused by irrelevant stimulation (Ferreira-Santos et al., 2012).

Accordingly, we measured the characteristic waveforms of N1 and P2 induced by standard and deviation stimuli in different implantation periods in the oddball paradigm. Besides, we compared the changes of the incubation period and amplitude at different implantation periods with the normal control group.

The third aim is to investigate MMN in CI users with the increase of implantation time. MMN is thought to represent the brain’s response to a mismatch of the current stimulus representation and a trace in short- or long-term auditory memory (Naatanen et al., 2011; Moberly et al., 2016). Nina Kraus et al. (1993) studied MMN in nine postlingual adult CI users with/da/and/ta/. They found MMN in eight good CI users but not in one poor user. They suggested that MMN has the potential to be a promising objective measure for the effect of CI. Previous research found that MMN was recorded in 80–85% of good performers but in only 15–20% of poor performers. In addition, patients with higher SIR scores showed a longer duration of MMN compared with those who had lower SIR scores (Shomeshwar Singh et al., 2004). Roman’s investigation showed that MMN was similar for well-performing CI users and normal hearing listeners in terms of speech perception, but abnormal or absent in poorly performing CI users (Roman et al., 2005).

As mentioned above, we used the area under MMN as the measurement of MMN, and the time point corresponding to half of the area is used as a measurement of MMN latency (Bishop and Hardiman, 2010; Moberly et al., 2016). We analyzed the changes in MMN amplitude and latency at different implantation periods and assessed the difference between CI users and healthy controls (HCs).

Fourth, we are interested in the changes in global brain functional connectivity during the recovery of CI users. A previous study found that in patients implanted with different kinds of cochlear implants, the results of behavioral tests showed consistency, but they showed differences in the functional connectivity of EEG (Maglione et al., 2017). The changes in EEG functional connectivity might better reflect the recovery of auditory function in patients with CI.

Consequently, we estimated functional connectivity using the phase lag index (PLI). By this way, we compared the difference in the EEG functional connectivity between CI users and HCs as the implantation time increased. The aim here was to find a functional connectivity measure sensitive to potential cochlear implant effects.

Overall, we measured the EEG response while patients with CI and HCs performed a speech recognition task in an oddball paradigm experiment. Using ERP analysis, we showed the time domain characteristic change in patients with CI as the implantation time increased. Using functional connectivity analysis, we demonstrated the remodeling of brain functional connectivity in patients with CI.

## Materials and Methods

### Participants

Fifteen HC participants (nine males, ranging from 22 to 30 years old) and 10 postlingual deafened CI users (five males, ranging from 19 to 52 years old) took part in the experiment. Participants in both groups were right-handed and had no history of neurological or psychiatric disorders, or any cognitive impairment. The CI users wound make an appointment to activate the device about a month after recovery from cochlear surgery. At this point, we would adjust the stimulation sound at different pure-tone frequencies to ensure the effectiveness of the device in the patient. The HC participants were age-matched with CI users (*P* = 0.19, see Table 1). Audiometry and otoscopy were carried out for the HC participants at enrolment. Pure-tone audiometry testing was used to measure the average of the pure-tone hearing threshold (0.5, 1, 2, and 4 kHz), also known as pure-tone average (PTA). PTA lower than 20 dB is defined as normal hearing. All participants in the normal group had normal hearing. The HC participants were examined in two recording sessions that were 2 months apart. For all CI users, speech perception tests were performed before cochlear implantation, when the device was activated, and 90/180 days after the device was activated. Tables 1, 2 show the demographic information for CI users and HC participants. The Medical Research Ethics Committee of Capital Medical University (Beijing, China) approved the study protocol in accordance with the recommendations of the Declaration of Helsinki for investigation of human participants. All participants provided written informed consent after being informed of the study details.

**TABLE 1:** Demographic information for HC subjects and CI users.

**Measure**	**HC (*n* = 15)**		**CI (*n* = 10)**		**Statistics**
	**Mean**	**SD**	**Mean**	**SD**	***P***
Age (years)	23.9	2.4	31.4	11	0.19
Gender (male)	9	n.a.	5	n.a.	0.77

*HC, healthy control; CI, cochlear implant.*

**TABLE 2:** Demographics of CI users.

**Subject**	**Gender**	**Age (years)**	**Ear implanted**	**CI experience (days)**	**Implant brand**	**Speech processing strategy**
CI1	M	33	R	180	MED-EL	FSP
CI2	M	41	R	180	MED-EL	FSP
CI3	F	24	L	180	MED-EL	FSP
CI4	F	31	R	180	MED-EL	FSP
CI5	M	43	R	180	MED-EL	FSP
CI6	F	52	L	180	MED-EL	FSP
CI7	F	21	L	180	MED-EL	FSP
CI8	M	21	L	180	MED-EL	FSP
CI9	F	29	L	180	MED-EL	FSP
CI10	M	19	L	180	MED-EL	FSP

*CI, cochlear implant; FSP, fine structure processing.*

### Speech Perception Test

Speech perception was evaluated by an audiologist who read the “Mandarin Vocabulary Adjacent Monosyllable Test Vocabulary” and “Mandarin Vocabulary Adjacent Two-syllable Test Vocabulary” to the participants by an amplifier equipment. The speech materials were presented at a comfortable level of 65 dB sound pressure level (SPL). Participants were asked to verbally repeat what they heard during the test. Eventually, the scores were calculated according to the percentage of words repeated properly. Furthermore, the speech perception tests were performed in CI users when the device was activated and 90 and 180 days after the device was activated.

### Stimuli and Task in the EEG/ERP Experiment

**FIGURE 1:**
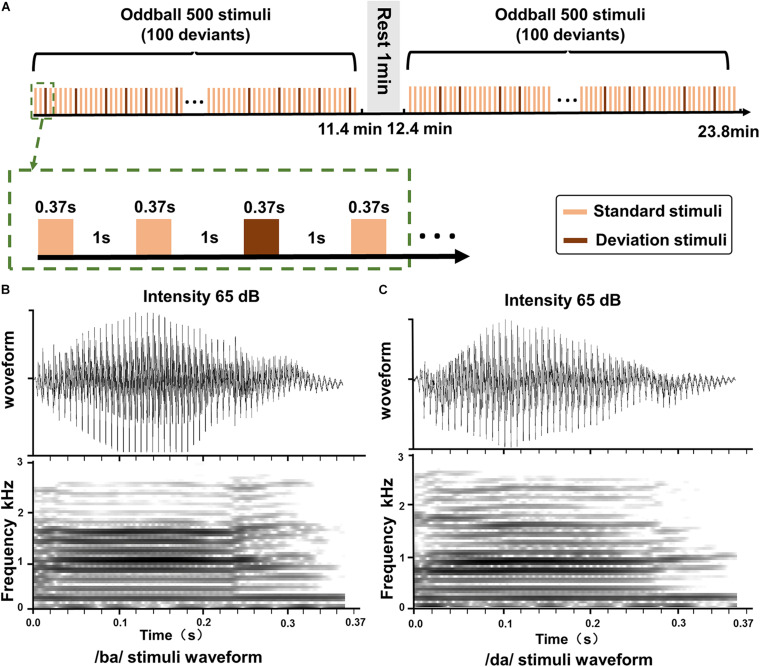
The oddball stimuli pattern and speech stimuli. **(A)** The standard stimulus and the deviation stimulus in the oddball paradigm. **(B)** The duration and intensity of the sound/ba/stimuli. **(C)** The duration and intensity of the sound/da/stimuli.

The oddball stimuli paradigm (Emmendorfer et al., 2020; Teixeira-Santos et al., 2020) with speech stimuli (350 ms duration with a 10-ms rise and fall time) was used in this study (Figure 1). The stimuli were delivered *via* E-Prime 2.0 (Psychology Software Tools, Inc.) and through two loudspeakers at both ears at a comfortable level of 65 dB SPL. The distance from the two loudspeakers to the participants was 100 cm. The two loudspeakers were positioned side to side and 45° to the ears. A standard stimulus/ba/was presented in 80% of the trials, together with a target stimulus/da/in 20% of the trials. There were a total of 1,000 auditory stimuli with 1,000 ms interstimulus intervals in the whole task.

### EEG Data Recording and Preprocessing

During the experiment, participants were instructed to sit on a comfortable chair and watch a silent movie clip in a shielded and sound-attenuated room. They were asked to ignore the auditory stimulus category information and avoid excessive eye and body movements. They were given a short break after 500 auditory stimuli to change body position and keep alert during the test. EEG was continuously recorded by a Geodesic EEG, Inc., (EGI) system through a precabled high-density 128-channel HydroCel Geodesic Sensor Net (HCGSN-128) referenced to the vertex. The sampling rate was 500 Hz and electrode impedances were kept at or below 5 kΩ according to the recommended value for the system. The recorded signals for all the electrodes were referenced to the vertex electrode (Cz).

Electroencephalogram data were processed using EEGLAB 12.02 Toolbox^1^ in MATLAB (R2013a; MathWorks, Inc.^2^ and custom scripts for batch processing. First, data were down-sampled to 250 Hz and filtered from 0.5 to 40 Hz using a short non-linear infinite impulse response filter. Second, the channel location file of the EGI system was imported. The EEG data were reviewed to delete bad channels. In order to reduce highly correlated signal from nearby electrodes, we down-sampled the data to the 10–10 international electrode system, resulting in 67 electrode channels (Xu et al., 2018; Furlong et al., 2020). Third, independent component analysis (ICA) was applied to the remaining data. Independent components (ICs) representing eyeblinks and heartbeats were visually identified and then removed from all datasets. The ICA procedure was done by the Infomax algorithm in EEGLAB. Afterward, these datasets were segmented into epochs from −200 to 700 ms relative to sound onset. The prestimulus interval (−200 to 0 ms) was used for baseline subtraction. Besides, the onset of CI stimulation evokes an electrical artifact and therefore inevitably corrupts the EEG signal. The CI artifact may largely be due to the radio frequency transmission of the signal to the receiver. Then, CI-artifact-related ICs were identified using the CI Artifact Correction (CIAC) algorithm, a plug-in in EEGLAB (Viola et al., 2009, 2011, 2012).

In our study, the CI-artifact-related ICs were selected both by CIAC and manual selection. The CI-artifact-related ICs selected by CIAC were not complete in some cases. Therefore, if the reconstruction of the individual ERPs was not reasonable after CIAC, the remaining CI-artifact-related ICs were selected manually based on the characteristics of the waveform and brain topographic maps of the ICs. The epochs contaminated by head movement were identified visually and rejected. After artifact rejection, about 700 EEG trials on average remained for standard stimuli and about 170 EEG trials on average remained for target stimuli.

### ERP Analysis

Event-related potentials were calculated by averaging epochs across participants for standard and target stimuli, respectively. The largest ERPs were found in the front-central region. The ERPs at FCz, C3, and C4 were also obtained for further analysis. Measurements of N1 and P2 peak amplitude were extracted for each participant and condition. N1 peak amplitude and latency were extracted as the minimum voltage in the 60–150-ms poststimulus interval. P2 peak and latency were extracted as the maximum voltage in the 150–250-ms (Roth et al., 1976) poststimulus interval at FCz, C3, and C4. Then, we calculated the brain topographic maps of N1 and P2 within ± 5 ms of the peaks.

### MMN Analysis

The difference in waveforms was also calculated between standard and target stimuli to obtain the MMN component. A valid MMN was identified when (1) a region of negativity was visibly confirmed from the differential waveform (deviant minus standard) and (2) its peak was consistent with the MMN topography (frontocentral negativity with reversals in lower temporal-posterior channels). For individuals, we performed the *t* test between the deviant and standard waveforms within the time window of 0–360 ms by sliding a 15-ms window with a step of 4 ms. The MMN onset was taken as the latency at which the *t* test (Bonferroni corrected for the number of executions) became significant. The MMN offset was taken as the latency at which the *t* test was no longer significant. Then, we calculated the area under the curve for the differential waveform (deviant minus standard) from the MMN onset to the MMN offset. The MMN area was used as a measurement of MMN magnitude. MMN latency was determined to be the time point that corresponded to the 50% of the area under the curve (Luck and Hillyard, 1990; Bishop and Hardiman, 2010; Moberly et al., 2016).

### PLI Functional Connectivity Analysis

We analyzed PLIs in the alpha (8–13 Hz) frequency bands with time period selected at 0–450 ms. We calculated the spectral density using fast Fourier transform, extracted phase information, calculated the phase difference between electrodes, and extracted the phase difference matrix. Subsequently, we made a correlation matrix from individual PLI values using a customized code in MATLAB (Kral et al., 2017; Ueda et al., 2020).

After preprocessing, we calculated EEG data with dimensions *E* × *T* × *S* (where *E* represents the number of electrodes, *T* represents the number of points in each segment, and *S* represents the number of segments). Each electrode pair had a PLI value at each time–frequency point. To calculate the PLI of electrode *i* and electrode *k*, we first obtain the *S* segment time–frequency information of the EEG signals by the short-time Fourier change

(1) $$X\,\left(n,\omega \right)=\sum_{\tau =-\infty }^{\infty }x\,\left(\tau \right)\,h\,\left(n-\tau \right)\,e^{-j\,w\,\tau }$$

where *x*(τ) indicates the input EEG signal at time *τ*, and *h*(τ) indicates the length Hamming window function. The window length we chose in our research was 0.4 s with a step size of 0.4 ms. *X*(*n*,ω) is the two-dimensional function of time and frequency. At this point, we have obtained *S* time–frequency matrices storing phase information. We then estimated the time series of phase differences across electrodes as:

(2) $$\Delta \,\phi_{i,k}\,\left(n\right)=\phi_{i}\,\left(n\right)-\phi_{k}\,\left(n\right)$$

where ϕ_*i*_(*n*) and ϕ_*k*_(*n*) indicate the phase value of the *i* electrode and the *k* electrode at time *n* (Nobukawa et al., 2019). Next, the functional connectivity was calculated as the consistency in the distribution of instantaneous phase differences Δϕ_*i*,*k*_(*n*) defined as:

(3) $$\text{PLI}=\left|\frac{1}{S}\,\sum_{s=1}^{S}\text{sig}\,h\,\left[\Delta \,\phi \,\left(n_{s}\right)\right]\right|$$

where the consistency of the phase difference of *S* segments was examined (Zhang et al., 2018; Nobukawa et al., 2019).

The PLI ranges between 0 and 1. If the PLI is 0, the two signals are either not coupled or are coupled with a phase difference centered at approximately 0 mod π. If the PLI is 1, the two signals are perfectly phase locked at a value of Δϕ, which is different from 0 mod π. When this non-zero phase locking is strong, the PLI will be large. However, it is worth noting that the PLI does not indicate which of the two signals is leading in phase (Zhang et al., 2018).

### Statistical Analysis

In each group [HC participants and CI users at three time points: as the device was activated (CI-1), 90 (CI-90), and 180 (CI-180) days after device activation], the ERP characteristics (including amplitude and latency) of the standard stimulus and deviation stimulus were tested by paired two-sample *t* test. One-way ANOVA analysis was performed separately on mean amplitude and mean latency on the factor group (HC, CI-1, CI-90, and CI-180). Besides, we performed the variance homogeneity test to test whether our data was variance homogeneous and *P* > 0.05 was considered homogeneous of variance. The least significant difference (LSD) method was used to correct multiple comparisons between pairs. These analyses were conducted on MMN, N1, and P2 components for both standard and target stimuli to detect any difference in amplitude and latency between HC participants and CI users at the three time points. A statistical significance level of 0.05 was considered significant, and statistical analyses were performed with SPSS 22.0.

The functional connection analysis between groups used the two-sample *t* test under GRETNA,^3^ and *P* < 0.005 was considered a significant difference.

## Results

### Behavioral Result

**FIGURE 2:**
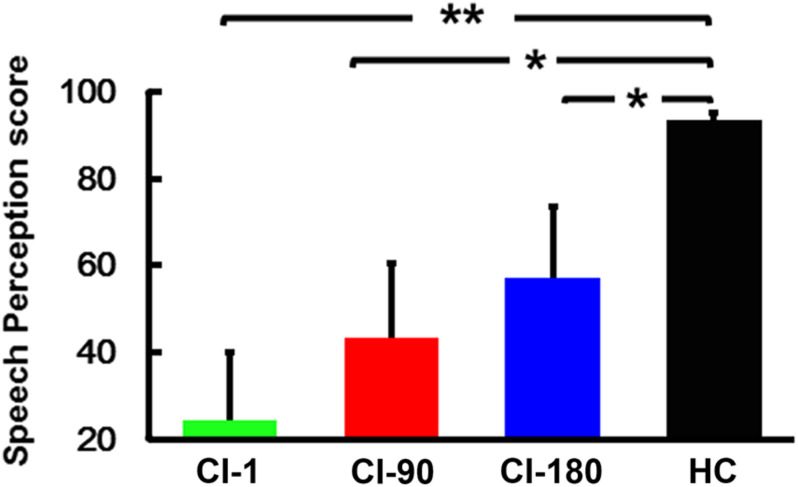
Speech perception score within groups, where the green histogram represents when the device is activated, the red histogram represents 90 days after device activation, the blue histogram represents 180 days after device activation, and the black histogram represents healthy controls. The vertical black line means standard deviation; *P < 0.05 and **P < 0.001.

The speech recognition scores (Figure 2) of the HCs (95.4 ± 4.6) were significantly higher than the speech recognition scores measured in CI users (CI-1: 23.4 ± 17.3, CI-90: 42.3 ± 19.1, and CI-180: 59.6 ± 17.5). The score of the CI users gradually approximated to the HCs as the implantation time increased. The results showed that as the implantation time increased, the speech recognition level of cochlear implanters gradually increased.

### ERP Components—Comparison of Standard Stimulus and Deviation Stimulus

**FIGURE 3:**
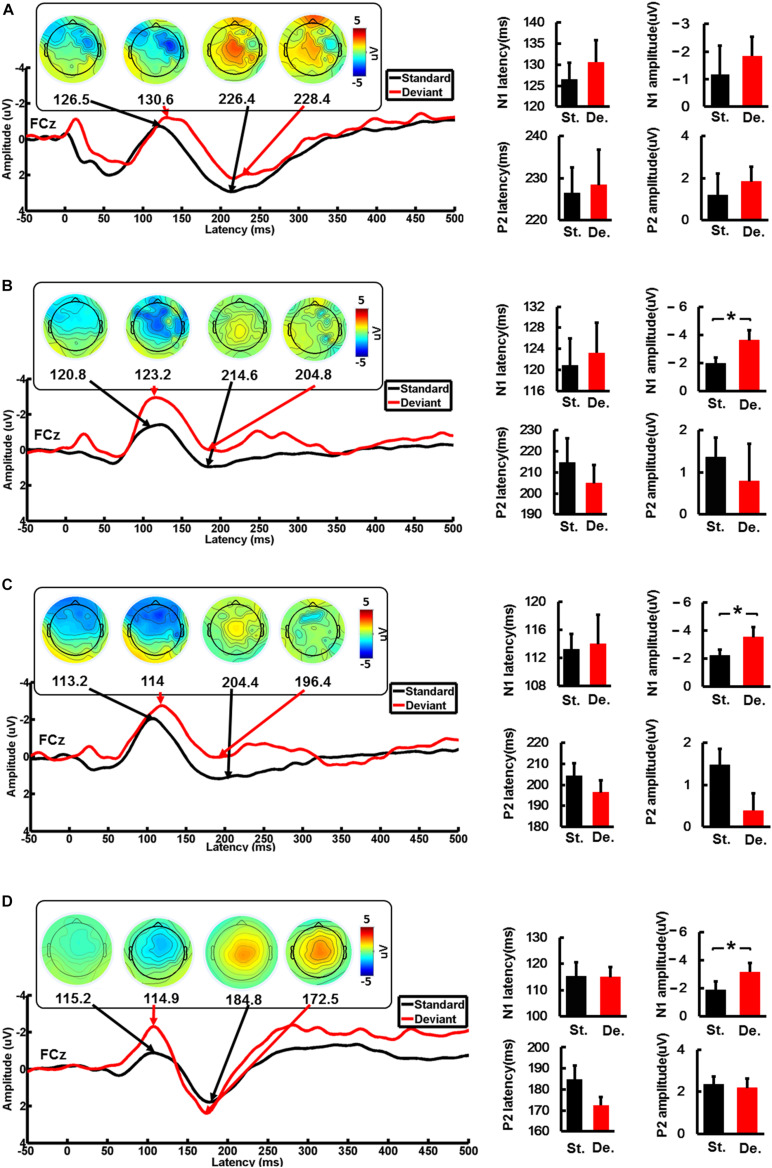
The average waveforms of the standard stimulus and the deviation stimulus at the FCz electrode. The topographic map of the N1 and P2 feature points, and the comparison of the amplitude and the latency of the standard stimulus and the deviation stimulus for CI users and the HC group. **(A)** When the device is activated, **(B)** 90 days after device activation, **(C)** 90 days after device activation, and **(D)** healthy controls. St., standard stimulus; De., standard stimulus. The vertical black line means standard error; *P < 0.05.

We analyzed the FCz ERPs induced by the standard stimulus and deviation stimulus in CI users when the device was activated, 90 days after device activation, and 180 days after device activation and the HC group. The changes in the latency and amplitude of N1 and P2 induced by standard stimulation and deviation stimulation in each group were compared. The ERP characteristics of the standard and the deviation stimulus did not show a significant difference in the CI-1 group. However, the amplitude of N1 showed a significant difference (*P* < 0.05) between standard and the deviation stimulus in the CI-90 (−1.9 ± 1.2 and −3.6 ± 2.4, *t* = 3.4, *P* = 0.008) and CI-180 (−2.2 ± 1.4 and −3.5 ± 2.4, *t* = 2.3, *P* = 0.046) groups. The difference was also shown in the HC group (−1.86 ± 0.9 and −3.2 ± 1.2, *t* = 6.9, *P* < 0.001) (Figure 3). Besides, the brain topographic maps of CI users were recovering gradually compared with the HCs.

### ERP Components—Comparison Between the CI Group and the HC Group

**FIGURE 4:**
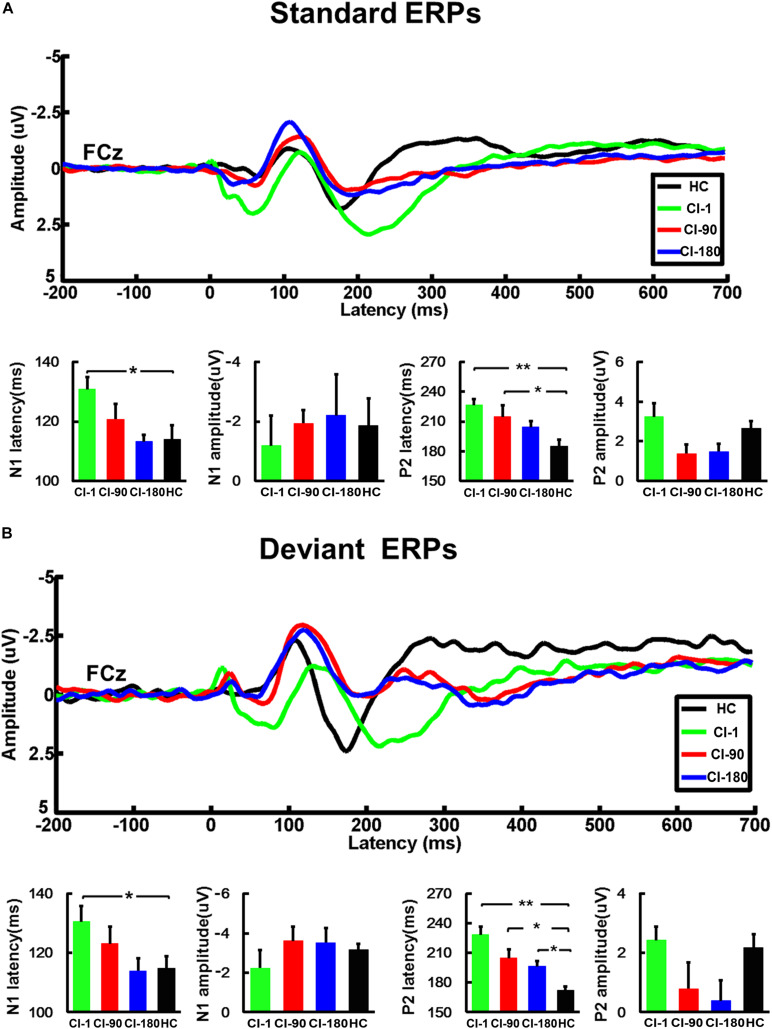
The average standard and deviant ERP waveforms of the FCz electrode and the comparison of the amplitude and the latency of the N1/P2 for CI users and the HC group. **(A)** The standard ERPs and **(B)** the deviant ERPs. The vertical black line means standard error; *P < 0.05 and **P < 0.001. CI-1, when the device is activated; CI-90, 90 days after device activation; CI-180, 180 days after device activation; HC, healthy controls.

We respectively analyzed the latency and amplitude of N1/P2 of FCz, C3, and C4 electrodes induced by standard and deviation stimuli. The results of FCz ERPs showed that the latency of N1/P2 in the CI group gradually approximated to the HC group as the implantation time increased regardless of standard stimulus or deviation stimulus (Figure 4). For the standard stimulus, the ANOVA analysis showed that there were significant differences in the latency of N1 (*F* = 2.9 and *P* = 0.04) and P2 (*F* = 5.1 and *P* = 0.004) among the four groups (CI-1, CI-90, CI-180, and HC). The results of N1 latency showed that CI-1 and HC were significantly different (*P* = 0.01), but CI-90 and CI-180 are not significantly different from HC. The results of P2 latency showed that CI-1 and HC and CI-90 and HC were significantly different (*P* < 0.001 and *P* = 0.01), but CI-180 is not significantly different from HC. For the deviation stimulus, the N1/P2 latency showed similar results except that CI-180 and HC were statistically different in P2 latency (*P* = 0.01). However, no similar result was found in the comparison of the amplitude of N1/P2.

**FIGURE 5:**
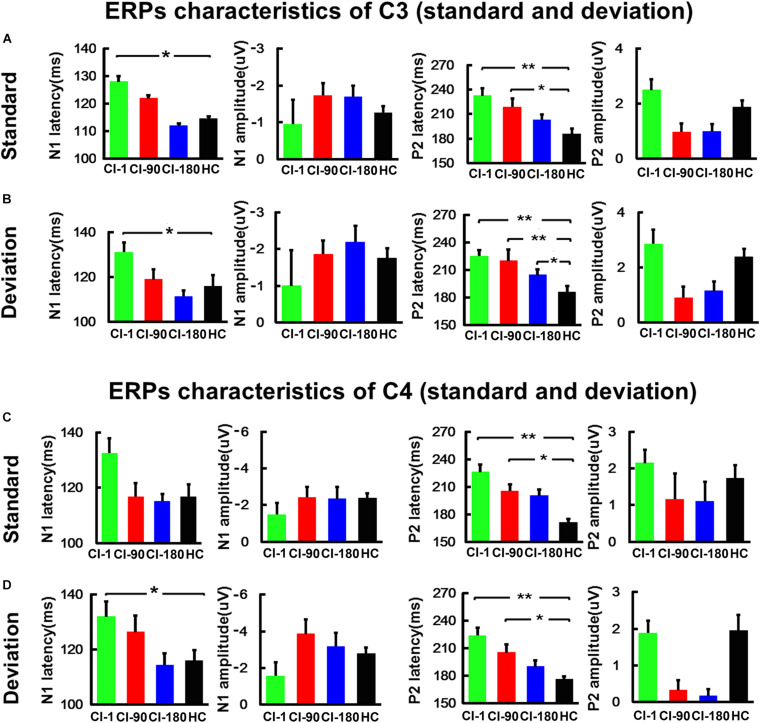
The comparison of the amplitude and the latency of the N1/P2 for CI users and the HC group at the C3 and C4 electrodes. **(A)** The comparison of the latency and amplitude of N1 and P2 induced by the standard stimulus at the C3 electrode. **(B)** The comparison of the latency and amplitude of N1 and P2 induced by the standard stimulus at the C4 electrode. **(C)** The comparison of the latency and amplitude of N1 and P2 induced by the deviation stimulus at the C3 electrode. **(D)** The comparison of the latency and amplitude of N1 and P2 induced by the deviation stimulus at the C4 electrode. The vertical black line means standard error; *P < 0.05 and **P < 0.001. CI-1, when the device is activated; CI-90, 90 days after device activation; CI-180, 180 days after device activation; HC, healthy controls.

Besides, for the ERPs of C3 (Figures 5A,B) and C4 (Figures 5C,D) electrodes, we did the same analysis as the FCz electrode. The results showed that the N1/P2 latency and amplitude characteristics of C3 and C4 electrodes are similar as those of the FCz electrode. The N1/P2 latency of CI users is longer than that of the HC group and approaches to the HC group regularly.

### MMN—Comparison Between CI Users and HC

**FIGURE 6:**
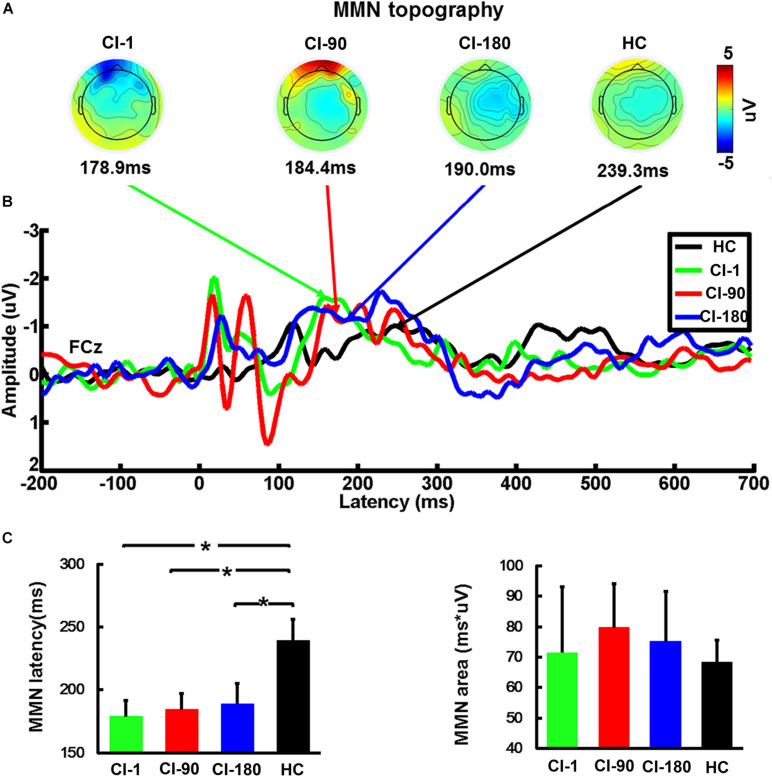
The average waveform of the FCz electrode MMN, the topographic map, and the comparison of the area and the latency of the MMN for CI users and HC. **(A)** The topographic map of MMN (latency ± 10 ms), **(B)** the average waveform of MMN at different implantation periods in the CI group and the HC group, and **(C)** the comparison of the area and the latency of the MMN for CI users and HC. The vertical black line means standard error; *P < 0.05. CI-1, when the device is activated; CI-90, 90 days after device activation; CI-180, 180 days after device activation; HC, healthy controls.

We analyzed the average MMN waveforms of the FCz electrodes (Figure 6B), and also we calculated the MMN area and latency corresponding to half of the area at the individual level. After that, we analyzed the topographic maps of the brain corresponding to the latency ±5 ms and compared the differences in the MMN area and latency between CI users and HCs in different periods by the one-way ANOVA test. It can be seen in Figure 6 that brain topographic maps of the MMN waveform in CI users with the device activated for 180 days were closer to those of NH participants (Figure 6A). The statistical results of the MMM area showed that CI users and healthy subjects did not show any difference, but the latency of MMN showed significant differences (*F* = 3.5 and *P* = 0.02) (Figure 6C). The CI-1, CI-90, and CI-180 groups contrasted with the HC group showed significant differences (*P* = 0.01, *P* = 0.017, and *P* = 0.028). Besides, the results also showed that the MMN latency of CI users approached to the HC group with the increase of implantation time.

### PLI Functional Connection

**FIGURE 7:**
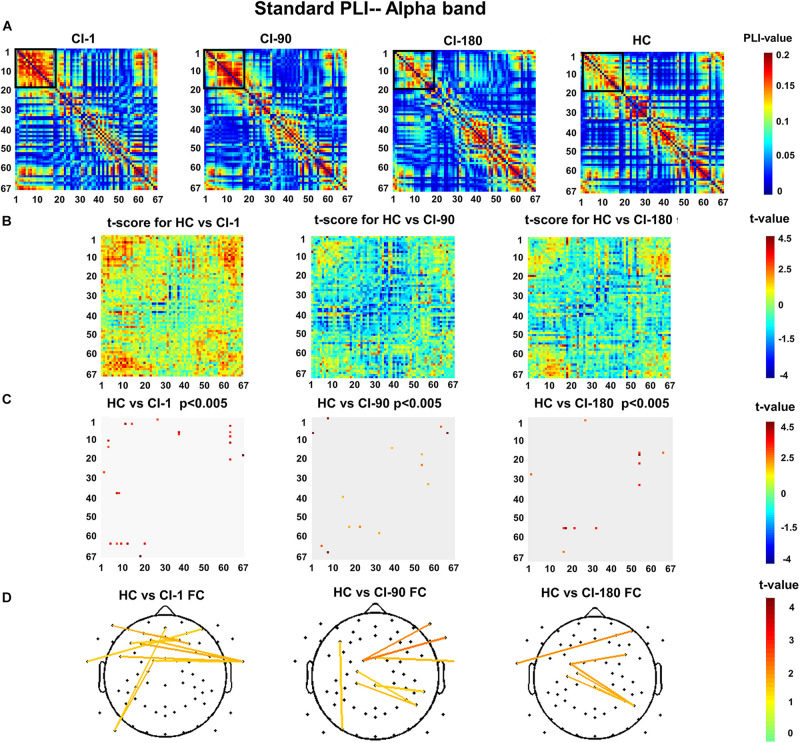
The standard stimulus function connection analysis **(A)** presents the values of the standard stimulus phase lag index (PLI) for 67 electrode pairs in the CI group and the HC group. **(B)** The *t* scores for differences between the HC and the CI groups and **(C)** adjusted for the false discovery rate *P* < 0.005. **(D)** The *t* scores adjusted for *P* < 0.005 and differential functional connection across the topography. The black box indicates the electrode pair area with the main difference. FC, functional connection.

Figures 7A,B show the standard stimulus PLI for the HC group and CI group and their differences. The two-sample *t* test revealed the significantly reduced PLI values for the CI group (Figure 7C). Significantly different electrode pairs between the CI group and the HC group gradually decreased as the implantation time increases, especially for several left and right auditory areas and frontal electrode pairs. The comparison result (Figure 7D) of the HC and CI-1 showed the significantly different electrode pairs in AF8–T9, F2–AF3, F2–F5, FCz–T10, FC1–P9, AFz–P9, F1–T10, F3–T10, F9–F4, and FT7–T10. The comparison result of the HC and CI-90 showed the significantly different electrode pairs in FC1–F10, FCz–T10, FC1–FT10, F5–PO7, C1–P6, CP1–P6, and CPz–TP8. The comparison result of the HC and CI-180 showed the significantly different electrode pairs in AF8–T9, FC3–P6, FC3–PC6, C1–P6, and C3–P6.

**FIGURE 8:**
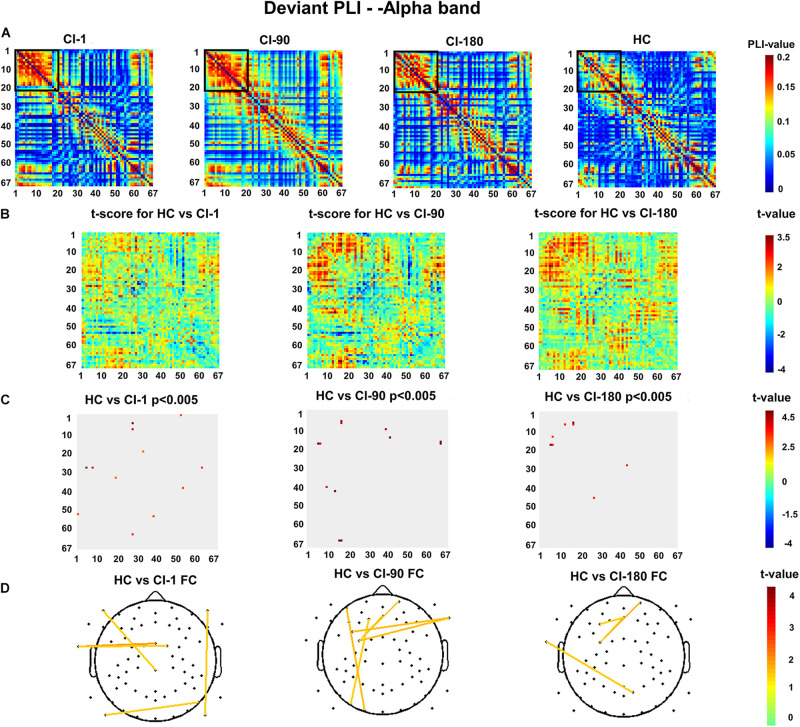
The deviant stimulus function connection analysis **(A)** presents the values of the deviant stimulus phase lag index (PLI) for 67 electrode pairs in the CI group and the HC group. **(B)** The *t* scores for differences between the HC and the CI groups and **(C)** adjusted for the false discovery rate *P* < 0.005. **(D)** The *t* scores adjusted for *P* < 0.005 and differential functional connection across the topography. The black box indicates the electrode pair area with the main difference. FC, functional connection.

Figures 8A,B show the deviant stimulus PLI for the HC group and CI group and their differences. Similarly, Figure 8C reveals the significantly reduced PLI values for the CI group. The comparison between the CI group and the HC group is also similar with the standard stimulus PLI. Significantly different electrode pairs (Figure 8D) between the CI group and the HC group were mainly for several left and right auditory areas and frontal electrodes. The comparison result of the HC and CI-1 showed the significantly different electrode pairs in F10–P10, FCz–T9, FC1–T9, P9–P8, and F9–CPz. The comparison result of the HC and CI-90 showed the significantly different electrode pairs in Fp2–FC3, Fz–FC3, F1–PO7, AF7–O1, and FC5–FT10. The comparison result of the HC and CI-180 showed the significantly different electrode pairs in Fp2–FC3, F3–Fz, FC3–Fz, and T9–PO4.

## Discussion

The present study examined the characteristics of ERPs/MMN and brain topographic maps in CI users after the CI-related artifacts were removed with ICA. Besides, we compared the difference in the EEG functional connection between CI patients and HCs. The ERP/MMN components and functional connection analysis used in this study may reflect some phases of auditory perception processes generated from auditory thalamocortical and corticocortical pathways such as primary and association cortices (Eggermont and Ponton, 2002; Ponton et al., 2002).

### Removal of CI-Related Artifacts

Artifact removal in the EEG recordings of CI users is of great significance in the study of auditory cortex functions. In agreement with previous studies, our results show that CI-related artifacts can be successfully reduced with ICA (Debener et al., 2008). Functional imaging methods such as functional MRI and PET are limited in their ability to study changes of neuronal function in CI users because of their safety and invasiveness, respectively (Anne Lise Giraud, 2009). Therefore, artifact removal with ICA enables the study of auditory cortex function to be more specific, and it may be of great clinical importance to use ERPs as an objective index of auditory cortical changes in CI users.

In our experiment, we first evaluated the speech recognition ability of CI users using the speech score scale and found that the implantation of cochlear implants can significantly improve the speech recognition ability of patients. However, for people who cannot be evaluated through behavioral assessments (such as people with prelingual deafness), we should find indicators to evaluate the effect of cochlear implants through the non-invasive scalp EEG test.

From this experiment, we found that several ERP indicators changed regularly during the recovery process of CI users and gradually approximated to the HC group as the implantation time increased.

### Characteristics of ERP Components

First, we found that the amplitude of N1 induced by the standard stimulus and the deviation stimulus was significantly different (*P* < 0.05; Figure 3D), which reflected the ability to recognize speech in the HC group. When the cochlear implant was implanted, there was no difference in the N1 amplitude induced by the standard stimulus and the deviation stimulus for CI users (Figure 3A), but after 90 days of adaptation, the N1 amplitude began to show a difference (Figure 3B), which also existed at 180 days (Figure 3C). The difference indicated that after 90 days of cochlear implantation, the speech recognition ability of CI users had improved. Here, we refer to the recovery of hearing abilities with CIs as a habilitation process, pointing out that the brain learns new strategies to adapt to the electrical input and thereby improves hearing. The improvement in speech perception was accompanied by an increase in their neural responses in the auditory cortex to complex tones. In fact, in HC participants, the target stimuli often elicit larger N1 than the standard stimuli (Näätänen and Picton, 1987), which is also consistent with our results. Previous studies showed that this N1 effect is caused by the habituation, refractoriness, or adaptation of neural populations, which is more sensitive to the change in stimuli properties (Horvath et al., 2008). The change of N1 in CI users may be caused by the enhancive neural synchrony owing to the reorganization of auditory cortical neurons. This reflects the gradual restoration of auditory nerve activity in CI users and makes it easy to increase the neural activity when processing target stimuli. Similarly, Sandmann et al. (2015) found that improvement in frequency discrimination and speech recognition was most pronounced over the first 8 weeks of CI experience. Also, they found a larger activation in the contralateral than in the ipsilateral auditory cortex, confirming the view that in CI users auditory information is predominantly processed in the contralateral auditory cortex, at least in the first year after implantation.

Second, we found that the N1/P2 latency of the standard stimulus and the deviation stimulus in the CI group was greater than that in the HC group. This indicates that CI patients are more sluggish than the HC group in processing target sounds (Han et al., 2016). More recent data suggest that the generators of N1 activity are located in the posterior face of the superior temporal sulcus (Picton et al., 1999). Contrary to the N1, the neural substrates of the P2 component are less well understood, and multiple sources have been implicated in its generation. These include the auditory output of the mesencephalic reticular activating system, the planum temporale, and Brodmann’s area 22 (Godey et al., 2001; Yvert et al., 2005). Besides, the latency of N1/P2 gradually decreases with the increase of implantation time, and it approaches the HC group. This also reflects the recovery effect of cochlear implantation. There was another study that conducted longitudinal research in cochlear implant users. Pantev et al. (2006) found that the increase of evoked brain activity over several months after implantation is the result of neural plasticity in the human auditory system, and within 180 days after implantation, CI patients may benefit most from postimplantation training. They also found that larger P1 and N1 amplitudes corresponded to a stronger perception in their MEG study.

### Characteristics of MMN Components

Third, our research found that the latency of the MMN of CI users was shorter than that of the HC group and there was a statistical difference (*P* < 0.05) in the stage of CI (CI-1, CI-90, and CI-180). However, the change of the latency of the MMN of CI users was gradually increasing, and the difference with the HC group was gradually decreasing (Figure 4C). Direct electrical stimulation of functional neural elements in the cochlea bypasses acoustic and mechanical transmission delays through the auditory periphery, which can account for a minor reduction in peak latencies (Don, 1995). MMN can reflect the ability of auditory perception and memory and is an important tool for auditory perception and memory in cognitive neuroscience research (Moberly et al., 2016). There are two major hypotheses about the generation of MMN: the adjustment mechanism and the adaptation mechanism. According to some studies, MMN depends on a memory trace created by the preceding stimuli. To be specific, the standard stimuli evoke neural responses that create a neural memory trace, and the MMN will be generated if the target stimuli arrive while the memory trace is still available (Zhang et al., 2011). Some studies have shown that standard and target stimuli can be different in terms of acoustic characteristics (intensity, frequency, duration, etc.) for MMN (Opitz et al., 2002; Doeller et al., 2003). Another mechanism recently put forward showed that MMN is generated from a much simpler local neuronal adaptation in the auditory cortex (Jääskeläinen et al., 2004). Here, we should pay attention to whether the duration of sound deprivation will affect the MMN response, as longer duration of sound deprivation might lose the auditory memory. A recent longitudinal study showed that within days after the initial switch-on of the implant, postlingually deaf CI users improved significantly in their hearing discrimination ability, and this relatively quick adaptation seems to take place even in individuals who experienced a long period of hearing deprivation (Sandmann et al., 2015; Stropahl et al., 2017). We supposed that the sound memory of the CI users will also be reconstructed with the improvement of speech recognition ability and this difference should be studied in future research. In our results, the MMN latency could be increased close to that in NH participants, which indicated the adaptation fits well with the increasing use of CI (Kelly et al., 2005; Alemi and Lehmann, 2019).

### PLI Functional Connectivity

Fourth, previous studies demonstrated that low-frequency EEG oscillations entrain to temporal modulations of sensory input. Different spatiotemporal characteristics of delta EEG encoding suggest that they potentially reflect different aspects of auditory motion processing (Bednar and Lalor, 2018). We analyzed the value of PLI in the alpha (8–13 Hz) band of CI users and HCs and calculated the difference in functional connection between CI users and HCs at different stages of implantation. Through statistical comparison, we found that the functional connection of the HCs was stronger than that of the CI users. Significant differences of electrode pairs mainly appear in the left and right auditory area and frontal electrodes, and the electrode pair gradually decreases as the implantation time increases. This result allows us to recognize the process of remodeling the auditory function of CI patients after CI more intuitively.

### Limitations

There are also some limitations to this study. First, the sample size should be increased and different types of speech stimuli should be used, since there is some variability in a small sample size of participants. In the PLI function connection analysis, we did not have the result of correction, but set the threshold of *P* at < 0.005, which may also be caused by the small amount of data. Second, the ERP components were only studied in CI users with the device activated for 180 days because of the limitation of time. For more information on the change of ERP characteristics, the time should be prolonged to 1–2 years or more after the CI device is activated. The third limitation we should consider is the generality of our EEG indicators. The principle of cochlear implants is that the CI increases auditory sensitivity by direct electrical activation of auditory nerve fibers, enabling phonemic awareness, discrimination, and identification, ultimately yielding speech understanding (Hossain et al., 2012). However, the various styles of cochlear implants with different manufacturers and algorithms (spectral peak, continuous interleaved sampler, advanced confined encoding, etc.) and the duration and intensity of stimuli might affect the results of data analysis. In this study, our task adopted the classic oddball paradigm, and the brands of cochlear implants are the same.

Besides, the applicability of multichannel EEG recordings might be more limited than a single-channel EEG acquisition, a faster and more comfortable technique, in a clinical setting (Mc Laughlin et al., 2013), but it allows the use of an ICA-based approach to attenuate the CI-related artifacts, providing a good solution for attenuating artifacts. Furthermore, multichannel EEG recordings could provide spatial information about the ERP with the brain topographic maps, which might be a better objective measure of CI performance together with the characteristics of an ERP waveform in the time domain.

### Conclusion

The behavioral performance revealed by speech perception scores showed a significant improvement with the increasing use of CI. The recovery of speech recognition ability can be evaluated by comparing the N1 amplitude of the standard stimulus and the deviation stimulus. In addition, the N1/P2/MMN latency characteristics and the PLI index in the alpha band can be used as the reference index for the remodeling of brain functional connectivity in CI patients and could be the objective index for the assessment of speech perception and the effects of cochlear implantation.

## Data Availability Statement

The original contributions presented in the study are included in the article/supplementary materials, further inquiries can be directed to the corresponding author/s.

## Ethics Statement

The studies involving human participants were reviewed and approved by the Medical Research Ethics Committee of Capital Medical University (Beijing, China). The patients/participants provided their written informed consent to participate in this study.

## Author Contributions

SW contributed to the study conception, design, data analysis and interpretation, statistical analyses, and manuscript writing. CL contributed to the conception, design, data acquisition and interpretation, and manuscript writing. ML and LS contributed to the conception, design, data acquisition and interpretation, and manuscript writing. XZ contributed to the conception, design, data acquisition and interpretation, and manuscript writing. All authors read and approved the final manuscript.

## Conflict of Interest

The authors declare that the research was conducted in the absence of any commercial or financial relationships that could be construed as a potential conflict of interest.

## Footnotes

1 https://sccn.ucsd.edu/eeglab/

2 https://www.mathworks.com/

3 https://www.nitrc.org/projects/gretna/
